# Interplay Between Childhood Maltreatment, Subclinical Post‐Traumatic Stress Symptoms, and IQ: Findings From the EU‐GEI Multicentre Case–Control Study

**DOI:** 10.1111/eip.70079

**Published:** 2025-08-09

**Authors:** Lucia Sideli, Monica Aas, Luis Alameda, Giulia Trotta, Daniele La Barbera, Caterina La Cascia, Laura Ferraro, Eva Velthorst, Giada Tripoli, Adriano Schimmenti, Andrea Fontana, Diego Quattrone, Charlotte Gayer‐Anderson, Victoria Rodriguez, Edoardo Spinazzola, Simona Stilo, Fabio Seminerio, Crocettarachele Sartorio, Giovanna Marrazzo, Antonio Lasalvia, Sarah Tosato, Ilaria Tarricone, Giuseppe D'Andrea, EU‐GEI WP2 Group, Silvia Amoretti, Álvaro Andreu‐Bernabeu, Grégoire Baudin, Stephanie Beards, Chiara Bonetto, Elena Bonora, Bibiana Cabrera, Angel Carracedo, Thomas Charpeaud, Javier Costas, Doriana Cristofalo, Manuel Durán‐Cutilla, Aziz Ferchiou, David Fraguas, Nathalie Franke, Flora Frijda, Cloe Llorente, Paz Garcia‐Portilla, Javier González Peñas, Kathryn Hubbard, Stéphane Jamain, Estela Jiménez‐López, Marion Leboyer, Gonzalo López Montoya, Esther Lorente‐Rovira, Covadonga M. Díaz‐Caneja, Camila Marcelino Loureiro, Maria Mayoral, Jessica Merchan, Dolores M. Moreno, Elles Messchaart, Ma Dolores Moltó, Gisela Mezquida, Carmen Moreno, Roberto Muratori, Nacher Juan, Mara Parellada, Marta Rapado‐Castro, Mirella Ruggeri, Jean‐Romain Richard, Rodríguez Solano, José Juan, Pilar A. Sáiz, Teresa Sánchez‐Gutierrez, Emilio Sánchez, Schürhoff Franck, Marco Seri, Rosana Shuhama, Fabian Termorshuizen, Anne‐Marie Tronche, Daniella van Dam, Elsje van der Ven, Celso Arango, Manuel Arrojo, Miguel Bernardo, Julio Bobes, Julio Sanjuán, Jose Luis Santos, Paulo Rossi Menezes, Cristina Marta Del‐Ben, Hannah E. Jongsma, Peter B. Jones, James B. Kirkbride, Pierre‐Michel Llorca, Andrea Tortelli, Baptiste Pignon, Lieuwe de Haan, Jean‐Paul Selten, Jim Van Os, Bart P. Rutten, Marta Di Forti, Robin M. Murray, Craig Morgan, Helen L. Fisher

**Affiliations:** ^1^ Department of Human Science LUMSA University Rome Italy; ^2^ Department of Psychosis Studies Institute of Psychiatry, Psychology & Neuroscience, King's College London London UK; ^3^ Department of Biomedicine, Neuroscience, and Advanced Diagnostic University of Palermo Palermo Italy; ^4^ Social, Genetic & Developmental Psychiatry Centre Institute of Psychiatry, Psychology & Neuroscience, King's College London London UK; ^5^ Centro Investigacion Biomedica en Red de Salud Mental (CIBERSAM), Instituto de Biomedicina de Sevilla (IBIS), Hospital Universitario Virgen del Rocio, Departamento de Psiquiatria Universidad de Sevilla Sevilla Spain; ^6^ Service of General Psychiatry, Treatment and Early Intervention in Psychosis Program Lausanne. University Hospital (CHUV) Lausanne Switzerland; ^7^ Mental Health Service Organization ‘GGZ Noord‐Holland‐Noord’, Department of Research Amsterdam the Netherlands; ^8^ Department of Health Promotion, Mother and Child Care, Internal Medicine and Medical Specialties University of Palermo Palermo Italy; ^9^ Department of Human and Social Science UKE—Kore University of Enna Enna Italy; ^10^ Department of Health Services and Population Research Institute of Psychiatry, Psychology & Neuroscience, King's College London London UK; ^11^ ESRC Centre for Society and Mental Health, King's College London London UK; ^12^ Department of Mental Health and Addiction Services, ASP Crotone Crotone Italy; ^13^ Section of Psychiatry, Department of Neuroscience, Biomedicine and Movement Sciences University of Verona Verona Italy; ^14^ Department of Medical and Surgical Sciences Alma Mater Studiorum—Bologna University Bologna Italy; ^15^ Department of Biomedical and NeuroMotor Sciences, Psychiatry Unit Alma Mater Studiorum—Bologna University Bologna Italy; ^16^ Institute of Psychiatry and Mental Health, Department of Child and Adolescent Psychiatry Hospital General Universitario Gregorio Marañón, School of Medicine, Universidad Complutense, ISGM, CIBERSAM Madrid Spain; ^17^ Department of Psychiatry, Psychiatric Genetic Group Instituto de Investigación Sanitaria de Santiago de Compostela, Complejo Hospitalario Universitario de Santiago de Compostela Santiago Spain; ^18^ Barcelona Clinic Schizophrenia Unit, Hospital Clinic, Departament de Medicina Institut de Neurociències (UBNeuro), Universitat de Barcelona (UB), Institut D'investigacions Biomèdiques, August Pi I Sunyer (IDIBAPS) Barcelona Spain; ^19^ Centro de Investigación Biomédica en Red de Salud Mental (CIBERSAM) Instituto de Salud Carlos III Madrid Spain; ^20^ Department of Medicine, Psychiatry Area, School of Medicine Universidad de Oviedo, ISPA, INEUROPA, Centro de Investigación Biomédica en Red de Salud Mental (CIBERSAM) Oviedo Spain; ^21^ Department of Psychiatry, School of Medicine Universidad de Valencia, Centro de Investigación Biomédica en Red de Salud Mental Valencia Spain; ^22^ Department of Psychiatry Hospital “Virgen de la Luz” Cuenca Spain; ^23^ Faculdade de Medicina Universidade de São Paulo São Paulo Brazil; ^24^ Faculdade de Medicina de Ribeirão Preto Universidade de São Paulo São Paulo Brazil; ^25^ PsyLife Group, Division of Psychiatry London UK; ^26^ Department of Psychiatry University of Cambridge Cambridge UK; ^27^ CAMEO Early Intervention Service Cambridgeshire and Peterborough National Health Service Foundation Trust Cambridge UK; ^28^ Université Clermont Auvergne Clermont‐Ferrand France; ^29^ Establissement Public de Santé, Maison Blanche Paris France; ^30^ AP‐HP, Groupe Hospitalier “Mondor” Pôle de Psychiatrie Créteil France; ^31^ Institut National de la Santé et de la Recherche Médicale Créteil France; ^32^ Fondation FondaMental Créteil France; ^33^ Early Psychosis Section Department of Psychiatry, Amsterdam UMC Amsterdam the Netherlands; ^34^ Institute for Mental Health, GGZ Rivierduinen Leiden the Netherlands; ^35^ Department of Psychiatry and Neuropsychology School for Mental Health and Neuroscience, Maastricht University Medical Centre Maastricht the Netherlands; ^36^ Department Psychiatry Utrecht University Medical Centre Utrecht the Netherlands

**Keywords:** childhood maltreatment, first episode, intelligence, post‐traumatic stress, psychosis

## Abstract

**Introduction:**

Evidence suggests that childhood maltreatment affects cognitive performance in both patients with psychosis and community controls. However, the interplay between childhood maltreatment, post‐traumatic stress symptoms (PTSS), and intelligence has not been investigated. This study investigated the relationship between childhood maltreatment, subclinical PTSS, and intelligence among patients with first‐episode psychosis (FEP) and community controls.

**Methods:**

Patients with FEP (*N* = 602) and controls (*N* = 853) from the EU‐GEI study were assessed for childhood maltreatment, PTSS, and intelligence quotient (IQ).

**Results:**

PTSS were associated with lower IQ among community controls but not among patients with FEP. In the FEP group, an interaction (*p* = 0.044) between PTSS and childhood maltreatment on IQ was found, such that the association between PTSS and lower IQ was only present among those exposed to childhood maltreatment. No interaction was evident in controls (*p* = 0.826).

**Conclusions:**

The findings suggest the relevance of cognitive rehabilitation for FEP patients with childhood maltreatment and PTSS.

## 1 Introduction

Accumulating research has consistently demonstrated that childhood maltreatment is associated with psychosis, but the mediators and moderators of this association are still unclear and suggest that cognition and post‐traumatic stress symptoms (PTSS) may play a role (Alameda et al. 2020; Sideli et al. 2020).

Findings on both general and clinical populations indicate that childhood maltreatment may impair cognitive performance (Rosa et al. 2023; Vargas et al. 2019), which is particularly detrimental for people suffering from psychosis as it reduces social and occupational functioning (Fares‐Otero et al. 2023; Montaner‐Ferrer et al. 2023). However, the association between childhood maltreatment and cognition is weaker among people with psychosis compared with controls (Dauvermann and Donohoe 2019; Vargas et al. 2019), arguably because of floor effects (Sideli et al. 2014; van Os et al. 2017). Furthermore, the impact of childhood maltreatment on cognition may vary by type of traumatic exposure. We previously found that both childhood abuse and neglect were associated with lower Intelligence Quotient (IQ) among controls without a psychotic disorder, whereas only childhood neglect was associated with lower IQ among people with first‐episode psychosis (FEP) (Sideli et al. 2022, 2023).

People with psychosis also report a greater prevalence of Post Traumatic Stress Disorders (PTSD) (Seong et al. 2023; Seow et al. 2016) and more frequent subclinical PTSS (Berry et al. 2013), that is PTSD symptoms not satisfying the clinical threshold for PTSD, according to established criteria (American Psychiatric Association 2022; World Health Organization 2019). PTSD and PTSS in psychosis may arise from childhood maltreatment (Bendall et al. 2008; Schäfer and Fisher 2011), adult life events (Beards et al. 2013; D'Andrea et al. 2023; van Zelst 2008) and the experience of psychosis itself (Buswell et al. 2021). Some community studies suggest that PTSS may increase the negative impact of childhood maltreatment on cognition (Burri et al. 2013; De Bellis et al. 2009, 2013). However, among people with psychosis, the association between PTSS and cognitive impairment has not been consistently replicated (Duke et al. 2010; Goodman et al. 2007; Peleikis et al. 2013). Furthermore, to the best of our knowledge, no study has examined the interplay between childhood maltreatment, PTSS and cognition in psychosis.

Using data from a large multi‐national study of psychosis, we sought to investigate the relationship between childhood maltreatment, subclinical PTSS and IQ among FEP patients and community controls. We hypothesised that: (1) compared to community controls, FEP patients would report a greater prevalence of PTSS; (2) PTSS would be associated with lower IQ in both patients and controls; and (3) PTSS would moderate the effect of childhood maltreatment on IQ in both groups, such that the association between exposure to maltreatment and lower IQ would be stronger among those with PTSS.

## 2 Methods

### 2.1 Sample

People with FEP and community controls participated in the EU‐GEI study, a multi‐centre incidence, case–control study carried out between May 2010 and April 2015 in five European countries and Brazil. The study was approved by the local Institutional Review Board and carried out in line with International Ethical Standards. Information about inclusion and exclusion criteria and sampling strategies was previously described (Gayer‐Anderson et al. 2020; van Os et al. 2008). The current study was conducted on a subset of 602 FEP and 853 controls with complete information about childhood maltreatment, PTSS and IQ (see Table S1 and Supporting Information 1).

### 2.2 Measures

Childhood maltreatment prior to age 18 was assessed using the Childhood Trauma Questionnaire (Bernstein et al. 1997). An overall ‘childhood maltreatment’ score, and separate ‘childhood abuse’ and ‘childhood neglect’ scores were calculated from the mean score of the respective items (rated 1 = never to 5 = very often). Consistent with previous studies (Sideli et al. 2022; van Os et al. 2017), three dichotomous variables for overall childhood maltreatment, abuse and neglect were then calculated using the 80th percentile of the control group as a cut‐off value (equal to 1.64, 1.40 and 2.00, respectively).

Presence of any PTSS in the week prior to study assessment was assessed in participants reporting any traumatic experience using the Impact of Events Scale‐6 (Thoresen et al. 2010) with two additional items to capture persistent re‐experiencing (‘I had dreams about it’ and ‘Pictures about it popped into my mind’) from the IES‐R (Weiss and Marmar 1997) (see Supporting Information). Items were rated on a 4‐point scale (0 = not at all to 4 = extremely), with total scores ranging from 0 to 32 (FEP: *α* = 0.86; controls: *α* = 0.89). To capture subclinical PTSS, we dichotomised the total score (0 = absent, 1+ = present).

IQ was assessed using an abbreviated version of the Wechsler Adult Intelligence Scale (WAIS‐III) (Blyler et al. 2000), including selected items of the following subtests: digit symbol coding (a measure of processing speed), arithmetic (working memory), block design (visuospatial processing), and information (verbal knowledge) (Velthorst et al. 2013, 2021).

Information about socio‐demographic characteristics (i.e., sex, age, country, education and ethnicity), lifetime cannabis use and current use of antipsychotics was collected using the MRC sociodemographic questionnaire (Mallet 1997), Cannabis Experience Questionnaire (Di Forti et al. 2014), and patients' clinical records.

### 2.3 Analyses

General linear regression models stratified for patients and controls were used to investigate the multiplicative interaction between childhood maltreatment and PTSS (independent dichotomous variables) on IQ (dependent continuous variable). Analyses were adjusted for the following covariates: sex, age, ethnicity (white vs. non‐white), education (from no education to post‐degree), study country, lifetime cannabis use (yes vs. no) and, only in the FEP group, current use of antipsychotics (none vs. one vs. > 1 antipsychotic medications). Results are reported as regression coefficients (B) and standardised betas (*β*). Analyses were carried out using SPSS v27.0.

## 3 Results

### 3.1 Associations Between Childhood Maltreatment, PTSS and IQ

Average IQ was significantly lower among FEP patients and prevalence of childhood maltreatment and PTSS were higher (Table S2). In both patients and controls, childhood maltreatment was associated with PTSS. In both groups, childhood maltreatment was associated with lower IQ, but PTSS was associated with lower IQ only in the control group (Table S3).

### 3.2 Interaction Between Childhood Maltreatment × PTSS on IQ

**FIGURE 1:**
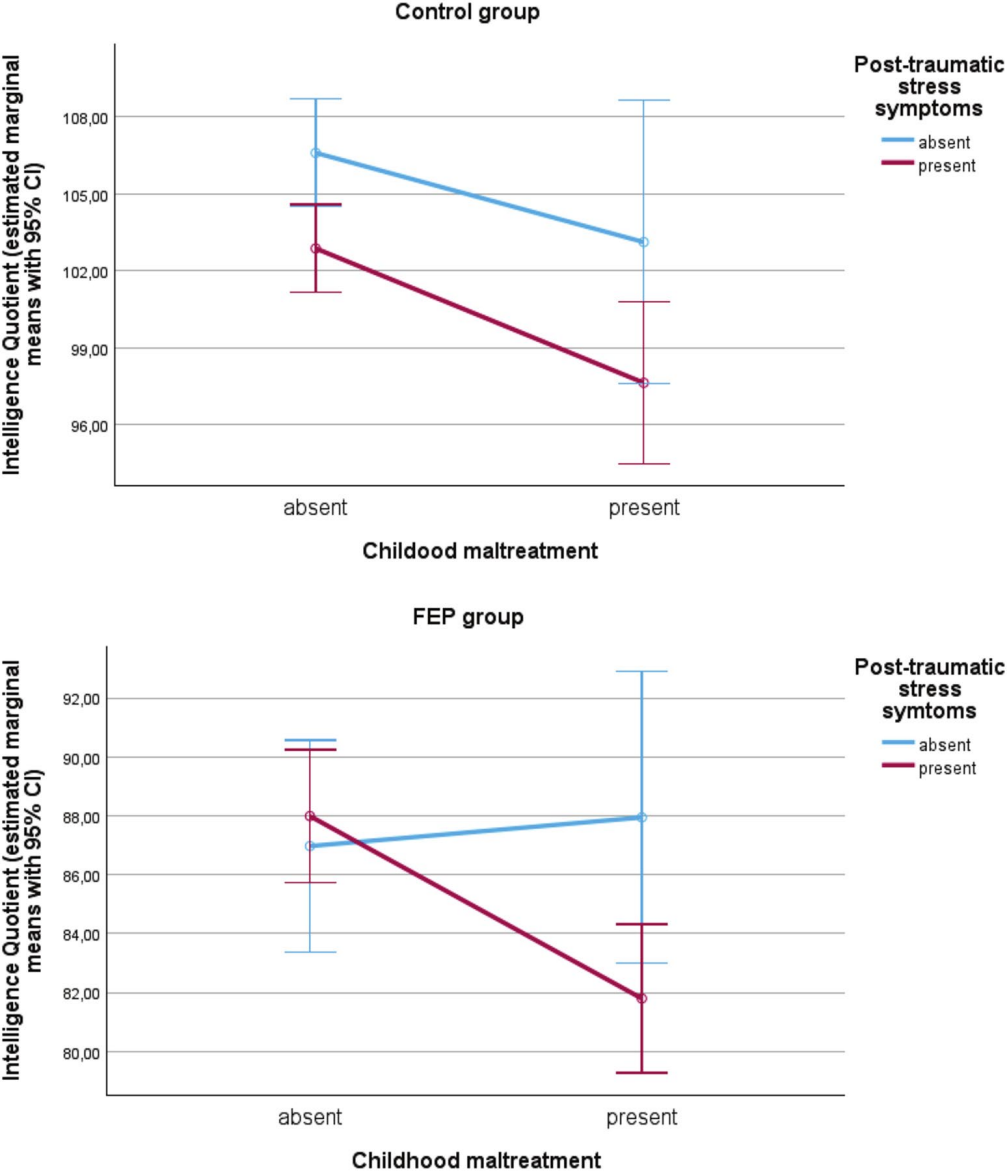
Interaction between childhood maltreatment and subclinical post‐traumatic stress symptoms. CI, confidence intervals; FEP, first‐episode psychosis.

An interaction between childhood maltreatment and PTSS on IQ was evident in the FEP group (Table 1 and Figure 1). Among patients exposed to childhood maltreatment, a lower IQ was found in those where PTSS was present (*M* = 81.80, SD = 15.87) versus absent (*M* = 87.94, SD = 21.97). No association was found in the corresponding unexposed group (PTSS+ *M* = 86.97, SD = 19.30 vs. PTSS− *M* = 87.99, SD = 18.62).

**TABLE 1:** Interaction between childhood maltreatment and subclinical PTSS on IQ.

	Crude model			Adjusted model^a^		
	*B*	95% CI	*p*	*B*	95% CI	*p*
Unstandardized models						
Controls	*N* = 853			*N* = 847		
Childhood maltreatment^b^	−3.48	−9.38; 2.43	0.248	−2.50	−7.71; 2.70	0.345
Subclinical PTSS^c^	**−3.72**	**−6.44; −1.01**	**0.007**	**−2.45**	**−4.90; 0.00**	**0.050**
Childhood maltreatment × subclinical PTSS	−1.76	−8.67; 5.15	0.617	0.68	−5.36; 6.71	0.826
FEP patients	*N* = 602			*N* = 597		
Childhood maltreatment^b^	0.97	−5.15; 7.09	0.755	2.78	−3.03; 8.59	0.348
Subclinical PTSS^c^	1.02	−3.22; 5.26	0.637	−0.96	−4.98; 3.07	0.641
Childhood maltreatment × subclinical PTSS	**−7.17**	**−14.16; −0.17**	**0.045**	**−6.73**	−**13.28; −0.18**	**0.044**
Standardised models						
Controls	*N* = 853			*N* = 847		
Childhood maltreatment^b^	−0.19	−0.52; 0.14	0.248	−0.14	−0.43; 0.15	0.345
Subclinical PTSS^c^	**−0.21**	**−0.36; −0.06**	**0.007**	**−0.14**	**−0.27; −0.00**	**0.050**
Childhood maltreatment × subclinical PTSS	−0.10	−0.48; 0.29	0.617	0.04	−0.30; 0.37	0.826
FEP patients	*N* = 602			*N* = 597		
Childhood maltreatment^b^	0.05	−0.28; 0.39	0.755	0.15	−0.17; 0.47	0.348
Subclinical PTSS^c^	0.06	−0.18; 0.29	0.637	−0.05	−0.27; 0.17	0.641
Childhood maltreatment × subclinical PTSS	**−0.39**	**−0.77; −0.01**	**0.045**	**−0.37**	**−0.72; −0.01**	**0.044**

*Note:* Significant associations (*p* ≤ 0.05) are shown in bold type.

Abbreviations: CI, confidence intervals; FEP, first‐episode psychosis; IQ, intelligence quotient; PTSS, post‐traumatic stress symptoms.

^a^ Adjusted for sex, age, ethnicity, education, study country, lifetime cannabis use and antipsychotic treatment (only in the FEP group).

^b^ Exposure to childhood maltreatment was defined as mean CTQ > 80th percentile of the control group; significant associations (*p* ≤ 0.05) are shown in bold type.

^c^ Presence of any subclinical post‐traumatic stress symptoms was defined as at least one item of the 8‐item Impact of Events Scale scored ≥ 1 on a scale of 0–4.

By contrast, among community controls, PTSS was associated with lower IQ, without interaction with childhood maltreatment (Table 1; Figure 1). In this group, the association was evident both in those exposed to childhood maltreatment (PTSS+ *M* = 97.64, SD = 17.05 vs. PTSS− *M* = 103.13, SD = 19.39) and in those unexposed (PTSS+ *M* = 102.88, SD = 17.53 vs. PTSS− *M* = 106.60, SD = 18.24).

### 3.3 Effect of Specific Types of Childhood Adversities

No childhood abuse × PTSS interaction on IQ was observed in either the FEP or control groups. Only in the FEP group was a childhood neglect × PTSS interaction detected (unadj. *B* = −8.82, *p* = 0.013; adj. *B* = −7.82, *p* = 0.018) (Tables S4 and S5).

In the control group there was an association between PTSS and lower IQ independent of and without interaction with childhood abuse (unadj. *B* = −3.36, *p* = 0.015; adj. *B* = −2.27, *p* = 0.069) and childhood neglect (unadj. *B* = −4.20, *p* = 0.002; adj. *B* = −2.87 *p* = 0.021). The findings were consistent when a measure of moderate to extreme PTSS symptoms was used (Supporting Information: Sensitivity analyses).

## 4 Discussion

This study found that subclinical PTSS were associated with lower IQ among community controls but not among patients with FEP. In the FEP group, an interaction between PTSS and childhood maltreatment was found, such that the association between PTSS and lower IQ was only present among those maltreated in childhood. No interaction was found among controls. The findings suggest that PTSS might be differently related to IQ among FEP patients and controls. In the FEP group, the lack of association between PTSS and IQ is consistent with some of the previous studies on PTSD and cognition (Duke et al. 2010; Peleikis et al. 2013). However, in this group, PTSS symptoms might modify the impact of childhood maltreatment on intelligence (Aas et al. 2014; Dauvermann and Donohoe 2019). Furthermore, among controls, the impact of PTSS on IQ seemed partially independent from the exposure to childhood maltreatment (Burri et al. 2013).

We found that the moderating effect of PTSS on IQ was only evident for neglect. Literature has suggested that, among people with psychosis, the impact of neglect on IQ might be more consistent than that of abuse (Aas et al. 2012; Garcia et al. 2016; Li et al. 2017; Mørkved et al. 2020). In line with studies revealing an association between childhood neglect, PTSS, and greater symptom severity in psychotic disorders (Cakir et al. 2016; Vogel et al. 2011), we speculate that PTSS might increase the detrimental impact of childhood neglect on cognition both directly, through memory impairment, and indirectly by amplifying emotion dysregulation (Liu et al. 2020) which in turn affects cognitive performance (Romano et al. 2015).

The findings of this study should be interpreted considering some limitations. First, consistent with previous literature (Vargas et al. 2019), childhood maltreatment could be considered to contribute to the lower IQ observed in FEP patients, which is affected by numerous factors, including genetic liability, developmental abnormalities, and social disadvantage. Second, although childhood maltreatment and PTSS were assessed with reference to non‐overlapping timeframes (i.e., up to age 18 for childhood maltreatment; over the past 7 days for PTSS), the retrospective study design prevents any inference about temporal order. Third, the sample size and missing data might have adversely affected the study's power to detect interactions. Fourth, the high prevalence of PTSS among FEP patients and their particularly low mean IQ might have obscured any potential association between PTSS and IQ in this group. In addition, the association of childhood maltreatment with lower IQ may involve other psychopathological factors (e.g., depression) not assessed here. Fifth, we were unable to include the PTSS score as a continuous variable in analyses as it was extremely skewed, thus limiting the conclusions that can be drawn. Finally, mediation analyses were not performed since we could not rule out that PTSS resulted from later life events, or the psychosis experience itself.

Nonetheless, these findings confirm the importance of adequately assessing (Carr et al. 2018; Redmond et al. 2025) and treating PTSD and subclinical PTSS among people with psychosis (Schäfer and Fisher 2011; Swan et al. 2017) and suggest an interplay between childhood maltreatment and PTSS on cognition (Christy et al. 2023; Rodriguez et al. 2021). If the results are replicated in larger, longitudinal samples, they could indicate the need for cognitive rehabilitation for FEP patients with childhood maltreatment histories and PTSS.

## Supporting information


**Data S1:** eip70079‐sup‐0001‐Supinfo.

## Data Availability

The data that support the findings of this study are available from the corresponding author upon reasonable request.

## Appendix A EU‐GEI WP2 Group

The European Network of National Schizophrenia Networks Studying Gene‐Environment Interactions (EU‐GEI) WP2 Group members includes: Silvia Amoretti, PhD, Barcelona Clinic Schizophrenia Unit, Hospital Clinic, Departament de Medicina, Institut de Neurociències (UBNeuro), Universitat de Barcelona (UB), Institut d'Investigacions Biomèdiques, August Pi I Sunyer (IDIBAPS), Barcelona, Spain; Centro de Investigación Biomédica en Red de Salud Mental CIBERSAM), Instituto de Salud Carlos III, Madrid, Spain; Álvaro Andreu‐Bernabeu, Child and Adolescent Psychiatry Department, Hospital General Universitario Gregorio Marañón, Universidad Complutense, Madrid, Spain; Grégoire Baudin, MSc, AP‐HP, Groupe Hospitalier ‘Mondor’, Pôle de Psychiatrie, Créteil, France, Institut National de la Santé et de la Recherche Médicale, U955, Créteil, France; Stephanie Beards, PhD, Department of Health Service and Population Research, Institute of Psychiatry, King's College London, London, England; Chiara Bonetto, PhD, Section of Psychiatry, Department of Neuroscience, Biomedicine and Movement Sciences, University of Verona, Verona, Italy; Elena Bonora, PhD, Department of Medical and Surgical Science, Genetic Unit, Alma Mater Studiorium Università di Bologna, Bologna, Italy; Bibiana Cabrera, MSc, PhD, Barcelona Clinic Schizophrenia Unit, Neuroscience Institute, Hospital Clinic of Barcelona, Barcelona, Spain, CIBERSAM, Spain; Angel Carracedo, MD, PhD, Fundación Pública Galega de Medicina Xenómica, Hospital Clínico Universitario, CIBERER, Santiago de Compostela, Spain; Thomas Charpeaud, MD, Fondation Fondamental, Créteil, France, CMP B CHU, Clermont Ferrand, France and Université Clermont Auvergne, Clermont‐Ferrand, France; Javier Costas, PhD, Fundación Pública Galega de Medicina Xenómica, Hospital Clínico Universitario, Santiago de Compostela, Spain; Doriana Cristofalo, MA, Section of Psychiatry, Department of Neuroscience, Biomedicine and Movement Sciences, University of Verona, Verona, Italy; Manuel Durán‐Cutilla, MD, Department of Child and Adolescent Psychiatry, Hospital General Universitario Gregorio Marañón, Institute of Psychiatry and Mental Health, IiSGM, School of Medicine, Universidad Complutense, Madrid, Spain; Aziz Ferchiou, MD, AP‐HP, Groupe Hospitalier ‘Mondor’, Pôle de Psychiatrie, Créteil, France, Institut National de la Santé et de la Recherche Médicale, U955, Créteil, France; David Fraguas, MD, PhD, Biomedical Research Networking Center for Mental Health Network (CIBERSAM), Barcelona, Spain; Department of Child and Adolescent Psychiatry, Hospital General Universitario Gregorio Marañón, Institute of Psychiatry and Mental Health, IiSGM, School of Medicine, Universidad Complutense, Madrid, Spain; Nathalie Franke, MSc, Department of Psychiatry, Early Psychosis Section, Academic Medical Centre, University of Amsterdam, Amsterdam, the Netherlands; Flora Frijda, MSc, Etablissement Public de Santé Maison Blanche, Paris, France; Cloe Llorente, MD, Department of Psychiatry, Hospital General Universitario Gregorio Marañón, School of Medicine, Universidad Complutense, Investigación Sanitaria del Hospital Gregorio Marañón (CIBERSAM), Madrid, Spain; Paz Garcia‐Portilla, MD, PhD, Department of Medicine, Psychiatry Area, School of Medicine, Universidad de Oviedo, ISPA, INEUROPA, CIBERSAM, Oviedo, Spain; Javier González Peñas, Hospital Gregorio Marañón (CIBERSAM), IiSGM, School of Medicine, Madrid, Spain; Kathryn Hubbard, Institute of Psychiatry, Psychology and Neuroscience, King's College London, London; Stéphane Jamain, PhD, Institut National de la Santé et de la Recherche Médicale, U955, Créteil, France, Faculté de Médecine, Université Paris‐Est, Créteil, France and Fondation Fondamental, Créteil, France; Estela Jiménez‐López, MSc, Department of Psychiatry, Servicio de Psiquiatría Hospital ‘Virgen de la Luz,’ Cuenca, Spain; Marion Leboyer, MD, PhD, AP‐HP, Groupe Hospitalier ‘Mondor,’ Pôle de Psychiatrie, Créteil, France, Institut National de la Santé et de la Recherche Médicale, U955, Créteil, France, Faculté de Médecine, Université Paris‐Est, Créteil, France and Fondation Fondamental, Créteil, France; Gonzalo López Montoya, PhD, Department of Child and Adolescent Psychiatry, Hospital General Universitario Gregorio Marañón, School of Medicine, Universidad Complutense, IiSGM (CIBERSAM), Madrid, Spain; Esther Lorente‐Rovira, PhD, Department of Psychiatry, School of Medicine, Universidad de Valencia, CIBERSAM, Valencia, Spain; Covadonga M. Díaz‐Caneja, Child and Adolescent Psychiatry Department, Hospital General Universitario Gregorio Marañón, Universidad Complutense, Madrid, Spain; Camila Marcelino Loureiro, MD, Departamento de Neurociências e Ciencias do Comportamento, Faculdade de Medicina de Ribeirão Preto, Universidade de São Paulo, São Paulo, Brasil and Núcleo de Pesquina em Saúde Mental Populacional, Universidade de São Paulo, São Paulo, Brasil; Maria Mayoral, Institute of Psychiatry and Mental health, Department of Child and Adolescent Psychiatry, Hospital General Universitario Gregorio Marañón, School of Medicine, Universidad Complutense, IIiSGM, CIBERSAM, Madrid, Spain; Jessica Merchan, Institute of Psychiatry and Mental health, Department of Child and Adolescent Psychiatry, Hospital General Universitario Gregorio Marañón, School of Medicine, Universidad Complutense, IIiSGM, CIBERSAM, Madrid, Spain; Dolores M. Moreno, Department of Child and Adolescent Psychiatry, Hospital General Universitario Gregorio Marañón, School of Medicine, Universidad Complutense, IiSGM (CIBERSAM), Madrid, Spain; Elles Messchaart, MSc, Rivierduinen Centre for Mental Health, Leiden, the Netherlands; Ma Dolores Moltó, Department of Genetics, University of Valencia, Campus of Burjassot, Biomedical Research Institute INCLIVA, Valencia, Spain, Centro de Investigacion Biomedica en Red de Salud Mental (CIBERSAM), Madrid, Spain; Gisela Mezquida, PhD, Barcelona Clinic Schizophrenia Unit, Hospital Clinic, Departament de Medicina, Institut de Neurociències (UBNeuro), Universitat de Barcelona (UB), Institut d'Investigacions Biomèdiques, August Pi I Sunyer (IDIBAPS), Centro de Investigación Biomédica en Red de Salud Mental CIBERSAM), Instituto de Salud Carlos III, Madrid, Spain; Carmen Moreno, MD, Department of Psychiatry, Hospital General Universitario Gregorio Marañón, School of Medicine, Universidad Complutense, IiSGM (CIBERSAM), Madrid, Spain; Roberto Muratori, MD, Department of Mental Health and Pathological Addiction, Local Health Authority, Bologna, Italy; Nacher Juan, Neurobiology Unit, Department of Cell Biology, Interdisciplinary Research Structure for Biotechnology and Biomedicine (BIOTECMED), Universitat de València, Valencia, Spain, Centro de Investigación Biomédica en Red de Salud Mental (CIBERSAM): Spanish National Network for Research in Mental Health, Madrid, Spain, Fundación Investigación Hospital Clínico de Valencia, INCLIVA, Valencia, Spain; Mara Parellada, MD, PhD, Department of Child and Adolescent Psychiatry, Hospital General Universitario Gregorio Marañón, School of Medicine, Universidad Complutense, IiSGM (CIBERSAM), Madrid, Spain; Marta Rapado‐Castro, PhD, Department of Child and Adolescent Psychiatry, Hospital General Universitario Gregorio Marañón, Institute of Psychiatry and Mental Health, IiSGM, School of Medicine, Universidad Complutense, Madrid, Spain; Mirella Ruggeri, MD, PhD, Section of Psychiatry, Department of Neuroscience, Biomedicine and Movement Sciences, University of Verona, Verona, Italy; Jean‐Romain Richard, MSc, Institut National de la Santé et de la Recherche Médicale, U955, Créteil, France and Fondation Fondamental, Créteil, France; Rodríguez Solano, José Juan, MD, Puente de Vallecas Mental Health Department, Hospital Universitario Infanta Leonor/Hospital Virgen de la Torre, Centro de Salud Mental Puente de Vallecas, Madrid, Spain; Pilar A. Sáiz, Department of Psychiatry, School of Medicine, University of Oviedo, CIBERSAM. Instituto de Neurociencias del Principado de Asturias, INEUROPA, Oviedo, Spain; Servicio de Salud del Principado de Asturias (SESPA), Oviedo, Spain; Teresa Sánchez‐Gutierrez, Department of Child and Adolescent Psychiatry, Hospital General Universitario Gregorio Marañón, School of Medicine, Universidad Complutense, IiSGM, CIBERSAM, C/Doctor Esquerdo 46, 28007 Madrid, Spain. Faculty of Health Science. Universidad Internacional de La Rioja (UNIR), Madrid, Spain; Emilio Sánchez, MD, Department of Psychiatry, Hospital General Universitario Gregorio Marañón, School of Medicine, Universidad Complutense, IiSGM (CIBERSAM), Madrid, Spain; Schürhoff, Franck, MD, PhD, AP‐HP, Groupe Hospitalier ‘Mondor,’ Pôle de Psychiatrie, Créteil, France, Institut National de la Santé et de la Recherche Médicale, U955, Créteil, France, Faculté de Médecine, Université Paris‐Est, Créteil, France and Fondation Fondamental, Créteil, France; Marco Seri, MD, Department of Medical and Surgical Science, Genetic Unit, Alma Mater Studiorium Università di Bologna, Bologna, Italy; Rosana Shuhama, PhD, Departamento de Neurociências e Ciencias do Comportamento, Faculdade de Medicina de Ribeirão Preto, Universidade de São Paulo, São Paulo, Brasil and Núcleo de Pesquina emSaúde Mental Populacional, Universidade de São Paulo, São Paulo, Brasil; Fabian Termorshuizen, PhD, Department of Psychiatry and Neuropsychology, School for Mental Health and Neuroscience, South Limburg Mental Health Research and Teaching Network, Maastricht University Medical Centre, Maastricht, the Netherlands and Rivierduinen Centre for Mental Health, Leiden, the Netherlands; Anne‐Marie Tronche, MD, Fondation Fondamental, Créteil, France, CMP B CHU, Clermont Ferrand, France and Université Clermont Auvergne, ClermontFerrand, France; Daniella van Dam, PhD, Department of Psychiatry, Early Psychosis Section, Academic Medical Centre, University of Amsterdam, Amsterdam, the Netherlands; Elsje van der Ven, PhD, Department of Psychiatry and Neuropsychology, School for Mental Health and Neuroscience,n and Teaching Network, Maastricht University Medical Centre, Maastricht, the Netherlands and Rivierduinen Centre for Mental Health, Leiden, the Netherlands.
