# Supplementary material for: Interplay Between Childhood Maltreatment, Subclinical Post‐Traumatic Stress Symptoms, and IQ: Findings From the EU‐GEI Multicentre Case–Control Study
Source: Early Interv Psychiatry. 2025 Aug 9;19(8):e70079. doi: 10.1111/eip.70079 (PMC12335189; doi:10.1111/eip.70079)
Supplement: Supplementary file 1 — Data S1: eip70079‐sup‐0001‐Supinfo. [file EIP-19-0-s001.docx]

**Supplementary Materials**

**Sideli *et al.* Interplay between childhood maltreatment, subclinical post-traumatic stress symptoms, and IQ: findings from the EU‑GEI multicentre case–control study**

**Supplementary Table 1: Demographic characteristics of FEP patients and controls**

| **Variable** | **FEP (n=** **602)**  ***n* (%)** | **Controls (n=** **853)**  ***n* (%)** | ***χ^2^*/ *t* (*df*)** | ***p*** |
| --- | --- | --- | --- | --- |
| **Sex, Male** | 360 (59.8) | 387 (45.4) | 29.42 (1) | <0.001 |
| **Age, *M* (*SD*)** | 31.11 (10.40) | 36.25 (13.09) | -8.01 (1453) | <0.001 |
| **Ethnicity, Non-white** | 213 (35.4) | 174 (20.4) | 40.42 (1) | <0.001 |
| **Education**   - **No qualification** - **Compulsory** - **Tertiary** - **Job related** - **University** - **Post-degree** | 128 (21.3)  122 (20.3)  143 (23.8)  62 (10.3)  70 (11.6)  77 (12.8) | 33 (3.9)  103 (12.1)  215 (25.2)  151 (17.7)  213 (25.0)  138 (16.2) | 144.78 (5) | <0.001 |
| **Country**   - **UK** - **Holland** - **Spain** - **France** - **Italy** - **Brazil** | 111 (20.7)  94 (17.7)  130 (24.5)  57 (10.7)  69 (13.0)  70 (13.2) | 278 (32.6)  131 (15.4)  173 (20.3)  80 (9.4)  104 (12.2)  87 (10.2) | 25.58 (5) | <0.001 |
| **Lifetime cannabis use** | 597  405 (67.8) | 849  452 (53.2) | 30.95 (1) | <0.001 |
| **Antipsychotic treatment**   - **More than one** - **One** - **None** | 554  99 (17.9)  122 (22.1)  333 (60.1) |  |  |  |
| **Diagnosis**   - **Non affective** - **Affective** | 601  434 (72.2)  167 (27.8) |  |  |  |

FEP: first-episode psychosis; df: degrees of freedom; M: mean; SD: standard deviation.

**Supplementary information 1: Study participants:**

Only 602 out of 829 (72.6%) FEP patients and 853 out of 1283 (66.5%) community controls from the original study assessing the relationship between childhood maltreatment and IQ (Sideli et al., 2022) had complete information about post-traumatic stress symptoms in the past week. Compared to the original sample, the current patients and control group reported a higher level of education (χ^2^ = 14.12, *p* = 0.015; χ^2^ = 16.50, *p* = 0.006, respectively) and a higher prevalence of lifetime cannabis use (χ^2^ = 4.46, p = 0.035; χ^2^ = 28.30, *p* < 0.001, respectively).

**Supplementary Table 2: Childhood maltreatment, IQ, and subclinical post-traumatic stress symptoms of FEP patients versus community controls**

| **Variable** | **FEP**  **(n=** **602)**  ***n* (%)** | **Controls**  **(n=** **853)**  ***n* (%)** | ***t / χ^2^*(df)** | ***p*** | **OR / b**  **[95% CI]** | ***p*** |
| --- | --- | --- | --- | --- | --- | --- |
| **Maltreatment exposure ***   - **Overall childhood maltreatment, n (%)** - **abuse, n (%)** - **neglect, n (%)** | 252 (41.9)  254 (42.2)  243 (40.4) | 163 (19.1)  168 (19.7)  147 (17.2) | 89.61 (1)  89.75 (1)  96.26 (1) | <0.001  <0.001  <0.001 | 3.05 [2.41, 3.86]  2.98 [2.36, 3.76]  3.25 [2.56, 4.14] | <0.001  <0.001  <0.001 |
| **IQ, *M* (*SD*)** | 85.76 (18.36) | 103.34 (17.99) | -18.21 (1453) | <0.001 | -0.95 [-0.94, -0.95] | <0.001 |
| **Presence of subclinical post-traumatic stress symptoms**** | 451 (74.9) | 536 (62.8) | 23.60 (1) | <0.001 | 1.77 [1.40, 2.23] | <0.001 |

B: unstandardized beta; CI: confidence intervals; df: degrees of freedom; FEP: first-episode psychosis; IQ: intelligence quotient; M: Mean; OR: odds ratio; SD: standard deviation; *Exposure to overall childhood maltreatment, and separately childhood abuse, and childhood neglect were defined as mean Childhood Trauma Questionnaire score >80^th^ percentile of the control group; ** Presence of any subclinical post-traumatic stress symptoms was defined as at least one item of the 8-item Impact of Events Scale scored ≥1 on a scale 0-4.

**Supplementary Table 3: Difference in mean IQ in relation to childhood maltreatment and subclinical post-traumatic stress symptoms among FEP patients and community controls**

| **Maltreatment exposure*** | **Unexposed**  ***mean IQ (SD)*** | **Exposed**  ***mean iq (SD)*** | ***t* (*df*)** | ***p*** |
| --- | --- | --- | --- | --- |
| ***Controls (N=853)*** |  |  |  |  |
| **Childhood maltreatment (690 vs. 163)**  **Abuse (685 vs. 168)**  **Neglect (706 vs. 147)** | 104.38 (17.90)  104.68 (18.17)  104.09 (17.76) | 98.99 (17.75)  97.91 (16.18)  99.75 (18.67) | 3.46 (851)  4.42 (851)  2.67 (851) | <0.001  <0.001  0.008 |
| **Presence of subclinical post-traumatic stress symptoms** (317 vs. 536)** | 106.16 (18.39) | 101.68 (17.55) | 3.54 (851) | <0.001 |
| ***FEP Patients (N=602)*** |  |  |  |  |
| **Childhood maltreatment (350 vs. 252)**  **Abuse (348 vs. 254)**  **Neglect (359 vs. 243)** | 87.70 (18.80)  86.47 (18.74)  87.49 (19.16) | 83.06 (17.43)  84.78 (17.83)  83.20 (16.84) | 3.08 (600)  1.12 (600)  2.83 (600) | 0.002  0.264  0.005 |
| **Presence of subclinical post-traumatic stress symptoms** (151 vs. 451)** | 87.30 (20.19) | 85.24 (17.71) | 1.20 (600) | 0.232 |

df: degrees of freedom; FEP: first-episode psychosis; IQ: intelligence quotient; M: Mean; SD: standard deviation; *Exposure to overall childhood maltreatment, and separately childhood abuse, and childhood neglect were defined as mean Childhood Trauma Questionnaire score >80^th^ percentile of the control group; ** Presence of any subclinical post-traumatic stress symptoms was defined as at least one item of the 8-item Impact of Events Scale scored ≥1 on a scale 0-4..

**Supplementary Table 4: Interaction between childhood abuse and subclinical PTSS on IQ**

| **Unstandardized models** | **Crude Model** | | | **Adjusted Model**^a^ | | |
| --- | --- | --- | --- | --- | --- | --- |
|  | *B* | *95% CI* | *p* | *B* | *95% CI* | *p* |
| **Controls** | *N*=853 | | | *N*=847 | | |
| **Childhood abuse ^b^**  **Subclinical PTSS^c^**  **Childhood abuse * subclinical PTSS** | -4.08  **-3.36**  -2.80 | -9.96; 1.80  **-6.07; -0.65**  -9.65; 4.06 | 0.174  **0.015**  0.424 | -2.82  -2.27  -0.09 | -8.01; 2.37  -4.72; 0.18  -6.13; 5.95 | 0.286  0.069  0.977 |
| **FEP Patients** | *N*=602 | | | *N*=597 | | |
| **Childhood abuse ^b^**  **Subclinical PTSS^c^**  **Childhood abuse * subclinical PTSS** | -0.07  -1.19  -1.96 | -6.20; 6.05  -5.50; 3.13  -8.98; 5.05 | 0.982  0.589  0.583 | 1.33  -3.36  -0.70 | -4.43; 7.08  -7.38; 0.66  -7.28; 5.88 | 0.651  0.102  0.835 |
| **Standardized models** |  | | |  | | |
|  | *β* | *95% CI* | *p* | *β* | *95% CI* | *p* |
| **Controls** | *N*=853 | | | *N*=847 | | |
| **Childhood abuse ^b^**  **Subclinical PTSS^c^**  **Childhood abuse * subclinical PTSS** | -0.23  **-0.19**  -0.16 | -0.55; 0.10  **-0.34; -0.04**  -0.54; 0.23 | 0.174  **0.015**  0.424 | -0.16  -0.13  -0.01 | -0.45; 0.13  -0.26; 0.01  -0.34; 0.33 | 0.286  0.069  0.977 |
| **FEP Patients** | *N*=602 | | | *N*=597 | | |
| **Childhood abuse ^b^**  **Subclinical PTSS^c^**  **Childhood abuse * subclinical PTSS** | -0.00  -0.07  -0.11 | -0.34; 0.33  -0.30; 0.17  -0.49; 0.28 | 0.982  0.589  0.583 | 0.07  -0.18  -0.04 | -0.24; 0.39  -0.40; 0.04  -0.40; 0.32 | 0.651  0.102  0.835 |

Note. CI: confidence intervals; FEP: first-episode psychosis; IQ: intelligence quotient; PTSS: post-traumatic stress symptoms. ^a^adjusted for sex, age, ethnicity, education, study country, lifetime cannabis use, and antipsychotic treatment (only in FEP group); ^b^Exposure to childhood abuse was defined as mean CTQ >80th percentile of the control group; significant associations (*p*<.05) are shown in bold type; ^c^Presence of any subclinical post-traumatic stress symptoms was defined as at least one item of the 8-item Impact of Events Scale scored ≥1 on a scale 0-4.

**Supplementary Table 5:** **Interaction between childhood neglect and subclinical PTSS on IQ**

| **Unstandardized models** | **Crude Model** | | | **Adjusted Model**^a^ | | |
| --- | --- | --- | --- | --- | --- | --- |
|  | *B* | *95% CI* | *p* | *B* | *95% CI* | *p* |
| **Controls** | *N*=853 | | | *N*=847 | | |
| **Childhood neglect ^b^**  **Subclinical PTSS^c^**  **Childhood neglect * subclinical PTSS** | -3.69  **-4.20**  -0.36 | -9.43; 2.05  **-6.90; -1.50**  -7.26; 6.53 | 0.208  **0.002**  0.918 | -3.60  **-2.87**  2.93 | -8.62; 1.42  **-5.30; -0.44**  -3.09; 8.95 | 0.160  **0.021**  0.340 |
| **FEP Patients** | *N*=602 | | | *N*=597 | | |
| **Childhood neglect ^b^**  **Subclinical PTSS^c^**  **Childhood neglect * subclinical PTSS** | 2.53  1.47  **-8.82** | -3.56; 8.61  -2.76; 5.70  **-15.79; -1.84** | 0.416  0.494  **0.013** | 3.51  -0.60  **-7.82** | -2.22; 9.24  -4.61; 3.40  **-14.33; -1.32** | 0.229  0.767  **0.018** |
| **Standardized models** |  | | |  | | |
|  | *β* | *95% CI* | *p* | *β* | *95% CI* | *p* |
| **Controls** | *N*=853 | | | *N*=847 | | |
| **Childhood neglect ^b^**  **Subclinical PTSS^c^**  **Childhood neglect * subclinical PTSS** | -0.21  **-0.23**  -0.02 | -0.52; 0.11  **-0.38; -0.08**  -0.40; 0.36 | 0.208  **0.002**  0.918 | -0.20  **-0.16**  0.16 | -0.48; 0.08  **-0.30; -0.03**  -0.17; 0.50 | 0.160  **0.021**  0.340 |
| **FEP Patients** | N=602 | | | N=597 | | |
| **Childhood neglect ^b^**  **Subclinical PTSS^c^**  **Childhood neglect * subclinical PTSS** | 0.14  0.08  **-0.48** | -0.19; 0.47  -0.15; 0.31  **-0.86; -0.10** | 0.416  0.494  **0.013** | 0.19  -0.03  **-0.43** | -0.12; 0.50  -0.25; 0.19  **-0.78; -0.07** | 0.229  0.767  **0.018** |

Note. CI: confidence intervals; FEP: first-episode psychosis; IQ: intelligence quotient; PTSS: post-traumatic stress symptoms. ^a^adjusted for sex, age, ethnicity, education, study country, lifetime cannabis use, and antipsychotic treatment (only in FEP group); ^b^Exposure to childhood neglect was defined as mean CTQ >80th percentile of the control group; significant associations (*p*<.05) are shown in bold type; ^c^Presence of any subclinical post-traumatic stress symptoms was defined as at least one item of the 8-item Impact of Events Scale scored ≥1 on a scale 0-4.

**Supplementary information 2: Modified version of the Impact of Events Scale**


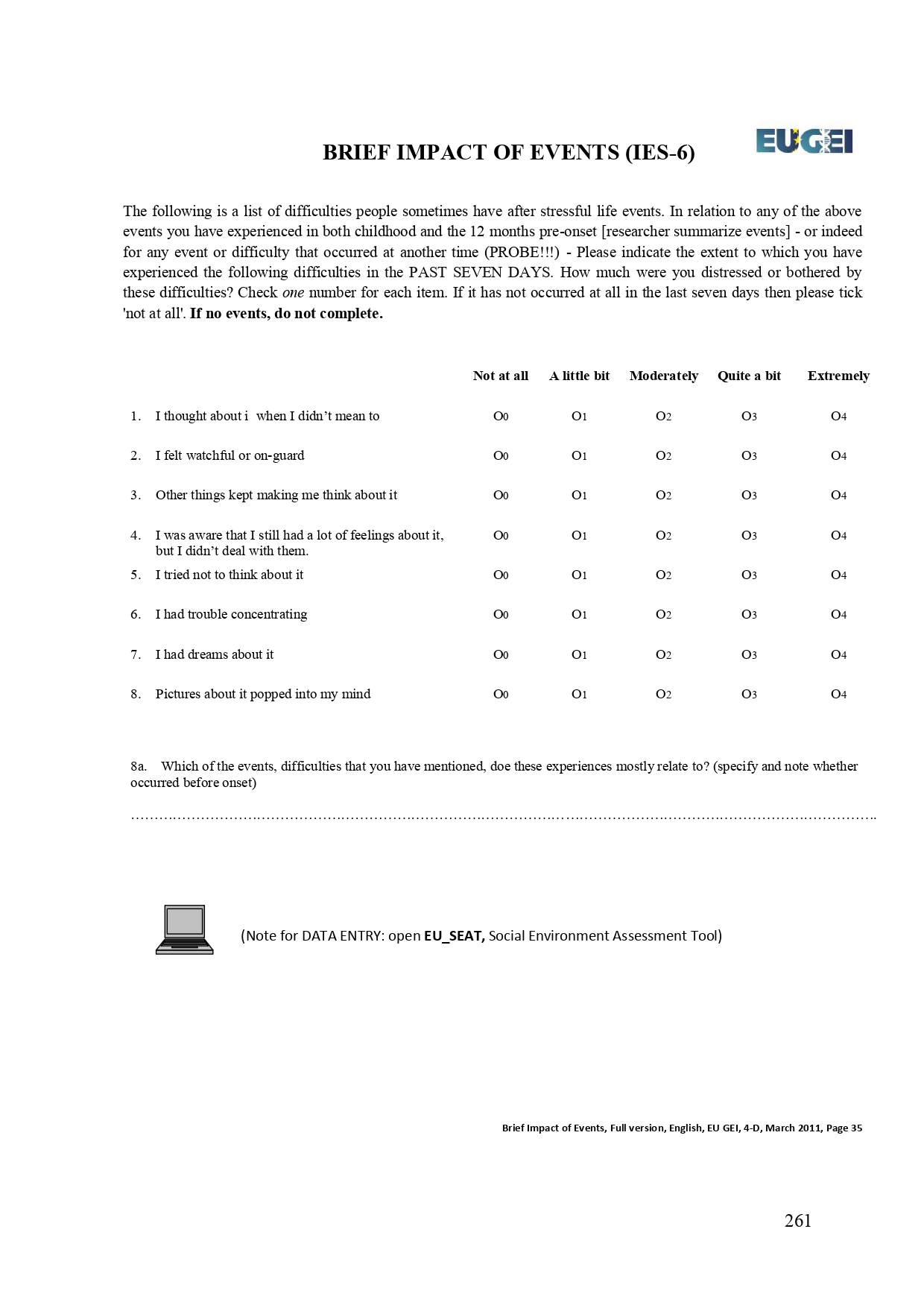


The instrument was administered in interview form by trained researchers after assessing traumatic experiences that occurred in childhood (i.e., the Childhood Traumatic Questionnaire (CTQ; Bernstein et al., 2003) and the Childhood Experience of Care and Abuse Interview (CECA; Bifulco et al., 2004), investigating about childhood abuse and childhood neglect occurring before age of 18) and in the past 12 months (i.e., the List of Threatening Experiences (LTE, Brugha et al., 1985), investigating about events such as serious illness or injury, loss of a family member, or separation from partner). If no traumatic experiences were reported, the Brief Impact of Events scale was not administered. If any traumatic experiences were reported, participants were asked to refer to this/these experience(s) in responding to the questions about post-traumatic stress symptoms (PTSS). Researchers took note of which experience(s) PTSS were related to. The above suggests that response to the Brief Impact of Events scale used in this study may reasonably reflect PTSS arising from early and recent traumatic events.

**Sensitivity analyses**

Sensitivity analyses were carried out using a dichotomous measure, defined as reporting at least one PTSS with a severity of 3 (quite a bit) or 4 (extremely) on a scale 0-4. The prevalence of one or more moderate to severe PTSS was 51.8% (312/602) among FEP patients and 35.4% (302/853) among controls. The univariate analyses found that the presence of more severe PTSS was associated with lower IQ among community controls (M=99.14 (SD=16.89) vs. M=105.65 SD=18.17), t=-5.13, p<0.001) but only to a non-significant degree among FEP patients (M=84.57 (SD=17.44) vs. M=87.04 (SD=19.26), t=-1.65, p=0.099).

**Supplementary Table 6: Interaction between childhood maltreatment and more severe PTSS on IQ**

| **Unstandardized models** | **Crude Model** | | | **Adjusted Model**^a^ | | |
| --- | --- | --- | --- | --- | --- | --- |
|  | *B* | *95% CI* | *p* | *B* | *95% CI* | *p* |
| **Controls** | *N*=853 | | | *N*=847 | | |
| **Childhood maltreatment ^b^**  **More severe PTSS^c^**  **Childhood maltreatment * more severe PTSS** | **-5.09**  **-6.34**  1.50 | **-9.20; -0.98**  -**9.16; -3.52**  -4.62; 7.63 | **0.015**  **<0.001**  0.630 | -3.34  **-3.79**  3.12 | -7.01; 0.34  **-6.36; -1.23**  -2.27; 8.52 | 0.075  **0.004**  0.257 |
| **FEP Patients** | *N*=602 | | | *N*=597 | | |
| **Childhood maltreatment ^b^**  **More severe PTSS^c^**  **Childhood maltreatment * more severe PTSS** | -0.21  1.42  **-7.27** | -4.78; 4.37  -2.44; 5.29  **-13.38; -1.17** | 0.929  0.471  **0.020** | 1.33  0.84  **-6.28** | -3.07; 5.72  -2.91; 4.59  **-12.02; -0.54** | 0.553 0.661  **0.032** |
| **Standardized models** |  | | |  | | |
|  | *β* | *95% CI* | *p* | *β* | *95% CI* | *p* |
| **Controls** | *N*=853 | | | *N*=847 | | |
| **Childhood maltreatment ^b^**  **More severe PTSS^c^**  **Childhood maltreatment * more severe PTSS** | **-0.28**  **-0.35**  0.08 | **-0.51; -0.06**  **-0.51; -0.20**  -0.26; 0.42 | **0.015**  **<0.001**  0.630 | -0.19  **-0.21**  0.17 | -0.39; 0.02  **-0.35; -0.07**  -0.13; 0.47 | 0.075  **0.004**  0.257 |
| **FEP Patients** | *N*=602 | | | *N*=597 | | |
| **Childhood maltreatment ^b^**  **More severe PTSS^c^**  **Childhood maltreatment * more severe PTSS** | -0.01  0.08  -**0.40** | -0.26; 0.24  -0.13; 0.29  **-0.73; -0.06** | 0.929  0.471  **0.020** | 0.07  0.05  **-0.34** | -0.17; 0.31  -0.16; 0.25  **-0.65; -0.03** | 0.553 0.661  **0.032** |

Note. CI: confidence intervals; FEP: first-episode psychosis; IQ: intelligence quotient; PTSS: post-traumatic stress symptoms. ^a^adjusted for sex. age. ethnicity. education. study country. lifetime cannabis use. and antipsychotic treatment (only in FEP group); ^b^Exposure to childhood maltreatment was defined as mean CTQ >80th percentile of the control group; ^c^Presence of more severe post-traumatic stress symptoms was defined as at least one item of the 8-item Impact of Events Scale scored ≥3 on a scale 0-4. Significant associations (*p*<.05) are shown in bold type.

**Supplementary Table 7: Interaction between childhood abuse and more severe PTSS on IQ**

| **Unstandardized models** | **Crude Model** | | | **Adjusted Model**^a^ | | |
| --- | --- | --- | --- | --- | --- | --- |
|  | *B* | *95% CI* | *p* | *B* | *95% CI* | *p* |
| **Controls** | *N*=853 | | | *N*=847 | | |
| **Childhood abuse ^b^**  **More severe PTSS^c^**  **Childhood abuse * more severe PTSS** | **-5.05**  **-5.46**  -1.27 | **-9.18; -0.91**  **-8.30; -2.62**  -7.31; 4.78 | **0.017**  **<0.001**  0.681 | -3.59  **-3.41**  1.83 | -7.30; 0.13  **-5.98; -0.83**  -3.54; 7.21 | 0.058  **0.010**  0.504 |
| **FEP Patients** | *N*=602 | | | *N*=597 | | |
| **Childhood abuse ^b^**  **More severe PTSS^c^**  **Childhood abuse * more severe PTSS** | -0.21  -1.51  -1.71 | -4.83; 4.41  -5.43; 2.41  -7.88; 4.46 | 0.930  0.449  0.587 | 1.55  -2.01  -0.89 | -2.78; 5.89  -5.74; 1.71  -6.64; 4.87 | 0.482  0.289  0.762 |
| **Standardized models** |  | | |  | | |
|  | *β* | *95% CI* | *p* | *β* | *95% CI* | *P* |
| **Controls** | *N*=853 | | | *N*=847 | | |
| **Childhood abuse ^b^**  **More severe PTSS^c^**  **Childhood abuse * more severe PTSS** | **-0.28**  **-0.30**  -0.07 | -**0.51; -0.05**  **-0.46; -0.15**  -0.41; 0.27 | **0.017**  **<0.001**  0.681 | -0.20  **-0.19**  0.10 | -0.41; 0.01  **-0.33; -0.05**  -0.20; 0.40 | 0.058  **0.010**  0.504 |
| **FEP Patients** | *N*=602 | | | *N*=597 | | |
| **Childhood abuse ^b^**  **More severe PTSS^c^**  **Childhood abuse * more severe PTSS** | -0.01  -0.08  -0.09 | -0.26; 0.24  -0.30; 0.13  -0.43; 0.24 | 0.930  0.449  0.587 | 0.08  -0.11  -0.05 | -0.15; 0.32  -0.31; 0.09  -0.36; 0.27 | 0.482  0.289  0.762 |

Note. CI: confidence intervals; FEP: first-episode psychosis; IQ: intelligence quotient; PTSS: post-traumatic stress symptoms. Note: Significant associations (p ≤ 0.05) are shown in bold type. ^a^adjusted for sex. age. ethnicity. education. study country. lifetime cannabis use. and antipsychotic treatment (only in FEP group); ^b^Exposure to childhood abuse was defined as mean CTQ >80th percentile of the control group; ^c^Presence of more severe post-traumatic stress symptoms was defined as at least one item of the 8-item Impact of Events Scale scored ≥3 on a scale 0-4. Significant associations (*p*<.05) are shown in bold type.

**Supplementary Table 8: Interaction between childhood neglect and more severe PTSS on IQ**

| **Unstandardized models** | **Crude Model** | | | **Adjusted Model**^a^ | | |
| --- | --- | --- | --- | --- | --- | --- |
|  | *B* | *95% CI* | *p* | *B* | *95% CI* | *p* |
| **Controls** | *N*=853 | | | *N*=847 | | |
| **Childhood neglect ^b^**  **More severe PTSS^c^**  **Childhood neglect * more severe PTSS** | -4.05  **-6.40**  0.42 | -8.12; 0.03  **-9.16; -3.65**  -6.01; 6.85 | 0.052  **<0.001**  0.899 | -3.17  **-3.94**  3.91 | -6.77; 0.43  **-6.43; -1.44**  -1.74; 9.57 | 0.085  **0.002**  0.175 |
| **FEP Patients** | *N*=602 | | | *N*=597 | | |
| **Childhood neglect ^b^**  **More severe PTSS^c^**  **Childhood neglect * more severe PTSS** | -0.33  0.77  **-6.58** | -4.84; 4.18  -3.03; 4.56  **-12.64; -0.52** | 0.885  0.692  **0.033** | 0.79  0.38  **-5.70** | -3.46; 5.04  -3.26; 4.03  **-11.33; -0.07** | 0.715  0.836  **0.047** |
| **Standardized models** |  | | |  | | |
|  | *β* | *95% CI* | *p* | *β* | *95% CI* | *p* |
| **Controls** | *N*=853 | | | *N*=847 | | |
| **Childhood neglect ^b^**  **More severe PTSS^c^**  **Childhood neglect * more severe PTSS** | -0.23  **-0.36**  0.02 | -0.45; 0.00  **-0.51; -0.20**  -0.33; 0.38 | 0.052  **<0.001**  0.899 | -0.18  **-0.22**  0.22 | -0.38; 0.02  **-0.36; -0.08**  -0.10; 0.53 | 0.085  **0.002**  0.175 |
| **FEP Patients** | N=602 | | | N=597 | | |
| **Childhood neglect ^b^**  **More severe PTSS**  **Childhood neglect * more severe PTSS** | -0.02  0.04  **-0.36** | -0.26; 0.23  -0.17; 0.25  **-0.69; -0.03** | 0.885  0.692  **0.033** | 0.04  0.02  **-0.31** | -0.19; 0.27  -0.18; 0.22  **-0.62; -0.00** | 0.715  0.836  **0.047** |

Note. CI: confidence intervals; FEP: first-episode psychosis; IQ: intelligence quotient; PTSS: post-traumatic stress symptoms. Note: Significant associations (p ≤ 0.05) are shown in bold type. ^a^adjusted for sex. age. ethnicity. education. study country. lifetime cannabis use. and antipsychotic treatment (only in FEP group); ^b^Exposure to childhood neglect was defined as mean CTQ >80th percentile of the control group; ^c^Presence of more severe post-traumatic stress symptoms was defined as at least one item of the 8-item Impact of Events Scale scored ≥3 on a scale 0-4. Significant associations (*p*<.05) are shown in bold type.

**References**

Bernstein, D. P., Stein, J. A., Newcomb, M. D., Walker, E., Pogge, D., Ahluvalia, T., Stokes, J., Handelsman, L., Medrano, M., Desmond, D., & Zule, W. (2003). Development and validation of a brief screening version of the Childhood Trauma Questionnaire. *Child Abuse & Neglect, 27*(2), 169–190. <https://doi.org/10.1016/s0145-2134(02)00541-0>

Bifulco, A., Brown, G. W., & Harris, T. O. (1994). Childhood Experience of Care and Abuse (CECA): a retrospective interview measure. *Journal of Child Psychology and Psychiatry, and allied Disciplines, 35*(8), 1419–1435. <https://doi.org/10.1111/j.1469-7610.1994.tb01284.x>

Brugha, T., Bebbington, P., Tennant, C., & Hurry, J. (1985). The List of Threatening Experiences: a subset of 12 life event categories with considerable long-term contextual threat. *Psychological Medicine, 15*(1), 189–194. https://doi.org/10.1017/s003329170002105x

Sideli, L., Schimmenti, A., La Barbera, D., La Cascia, C., Ferraro, L., Aas, M., Alameda, L., Velthorst, E., Fisher, H. L., Caretti, V., Trotta, G., Tripoli, G., Quattrone, D., Gayer-Anderson, C., Seminerio, F., Sartorio, C., Marrazzo, G., Lasalvia, A., Tosato, S., Tarricone, I., … EU-GEI WP2 Group (2022). Childhood Maltreatment, Educational Attainment, and IQ: Findings From a Multicentric Case-control Study of First-episode Psychosis (EU-GEI). *Schizophrenia Bulletin*, *48*(3), 575–589. https://doi.org/10.1093/schbul/sbac004
